# Discriminatory Precision of Renal Angina Index in Predicting Acute Kidney Injury in Children; a Systematic Review and Meta-Analysis

**Published:** 2020-03-26

**Authors:** Arash Abbasi, Pardis Mehdipour Rabori, Ramtin Farajollahi, Kosar Mohammed Ali, Nematollah Ataei, Mahmoud Yousefifard, Mostafa Hosseini

**Affiliations:** 1 Pediatric Chronic Kidney Disease Research Center, Tehran University of Medical Sciences, Tehran, Iran.; 2 Department of Pediatrics, Division of Nephrology, Children’s Medical Center, Tehran University of Medical Sciences, Tehran, Iran.; 3 Student Research Committee, Iran University of Medical Sciences, Tehran, Iran.; 4 College of Medicine, University of Sulaimani, Sulaimani, Iraq.; 5 Physiology Research Center, Iran University of Medical Sciences, Tehran, Iran.; 6 Department of Epidemiology and Biostatistics, School of Public Health, Tehran University of Medical Sciences, Tehran, Iran.

**Keywords:** Acute Kidney Injuries, Renal Insufficiency, Severity of Illness Index, Child

## Abstract

**Introduction:**

There is still controversy over the value of renal angina index (RAI) in predicting acute renal failure (AKI) in children. Therefore, the present study aims to provide evidence by conducting a systematic review and meta-analysis on the value of RAI in this regard.

**Methods:**

An extensive search of Medline, Embase, Scopus and Web of Science databases was conducted by the end of January 2020 using words related to RAI and AKI. Two independent reviewers screened and summarized the related studies. Data were analysed using STATA 14.0 statistical program and discriminatory precision of RAI was assessed.

**Results:**

Data from 11 studies were included. These studies included data from 3701 children (60.41% boys). There were 752 children with AKI and 2949 non-AKI children. Pooled analysis showed that the area under the ROC curve of RAI in prediction of AKI was 0.88 [95% confidence interval (CI): 0.85 to 0.91]. Sensitivity and specificity of this tool in predicting AKI were 0.85% (95% CI: 0.74% to 0.92%) and 0.79% (95% CI: 0.69% to 0.89%), respectively. The diagnostic odds ratio of RAI was 20.40 (95% CI: 9.62 to 43.25).

**Conclusion:**

The findings of the present meta-analysis showed that RAI is a reliable tool in predicting AKI in children.

## Introduction

Acute kidney injury (AKI) is a serious problem in children and adolescents and can rapidly progress to chronic kidney disease and result in the need for dialysis if not diagnosed in a timely manner. The prevalence of acute renal failure indicates that approximately 10% of children admitted to intensive care units develop AKI (1). The effect of this failure on mortality is significant (1, 2). Unfortunately, the onset and progression of AKI is often asymptomatic and its diagnosis is mainly based on functional biomarkers such as serum creatinine. But in recent years, due to the limitations of creatinine, researchers are seeking an alternative method (1, 3, 8).

Currently, several diagnostic methods for identifying children with kidney disease are available, but none of them provide a correct picture in the early stages of the disease. Meanwhile, the use of scoring systems such as the renal angina index (RAI) has received much attention in recent years. RAI was first introduced by Basu et al. in 2014 to improve the prediction of AKI in critically ill children (9). This study has two phases of derivation and validation. The findings of this study suggest that RAI is an acceptable and simple criterion for identifying children at risk for AKI. Recent research suggests that RAI can diagnose AKI and predict the patient's outcome. But there is still disagreement between studies and there is no consensus (10, 11). Therefore, the present study aims to provide evidence by conducting a systematic review and meta-analysis on the value of RAI in predicting AKI in children.

## Methods

- **Study design**

The present study is a systematic review and meta-analysis on the diagnostic value of RAI in predicting AKI in children. The study was designed based on meta-analysis of observational studies in epidemiology (MOOSE) statement (12).

- **Search strategy**

In the first step, the keywords associated with AKI and RAI were identified. Then by combining the related keywords using standard tags and Boolean operators for each database, a systematic search was performed on Medline, Embase, Scopus, and Web of Science electronic databases until the end of January 2020. The search query used in Medline database is reported in Appendix 1. To find additional articles or unpublished data, a manual-search was performed in the bibliography of the relevant studies, Google and Google Scholar. Applying this strategy resulted in the addition of two articles to the present study.

- **Selection criteria**

In the present study, diagnostic accuracy studies performed on renal angina index in diagnosis of acute renal failure in children were included. Inclusion criteria were confirmation of AKI via one of the standard methods, sensitivity and specificity (from the article or by contacting the authors) being provided or true positive (TP), true negative (TN)), false positive (FP) and false negative (FN) being provided. Both retrospective and prospective studies were included. Exclusion criteria were as follows: review studies, studies on adults, duplicate studies (use of same dataset in two studies), and lack of non-AKI group.

- **Data collection and quality assessment**

After combining the search records and eliminating duplicates, two independent reviewers screened the abstracts and selected potentially relevant studies. Then, they assessed and summarized the full-text of eligible studies. In case of disagreement, a third reviewer evaluated the findings and existing disagreement was resolved through discussion. Extracted data included first author’s name, year of publication, country, demographic data of patients (age, sex), sample size, standard criterion for defining AKI, severity of AKI, RAI evaluation time, cut-offs used for RAI and finally sensitivity, specificity, TP, TN, FP, and FN.

The risk of bias was assessed using guidelines proposed in Quality Assessment of Diagnostic Accuracy Studies 2 (QUADAS-2) (14).

- **Statistical analysis**

Statistical analyses were performed using STATA version 14.0 (Stata Corporation, College Station, TX). All studies were summarized and categorized based on sensitivity and specificity or TP, TN, FP, and FN. Then, the discriminatory power of RAI in predicting children's AKI was calculated using the “*midas” *command, which is a bivariate mixed-effects binary regression modelling framework. Results were reported as area under the summary receiver operating characteristics (SROC) curve (AUC) with 95% confidence interval (CI). In addition, sensitivity, specificity, negative and positive likelihood ratios and diagnostic odds ratio were calculated. Heterogeneity between studies was assessed using I2 test and p value less than 0.1 was considered significant (indicating Heterogeneity). Publication bias across studies was also assessed using Deek's funnel plot asymmetry test.

## Results

- **Study characteristics**

**Figure 1:**
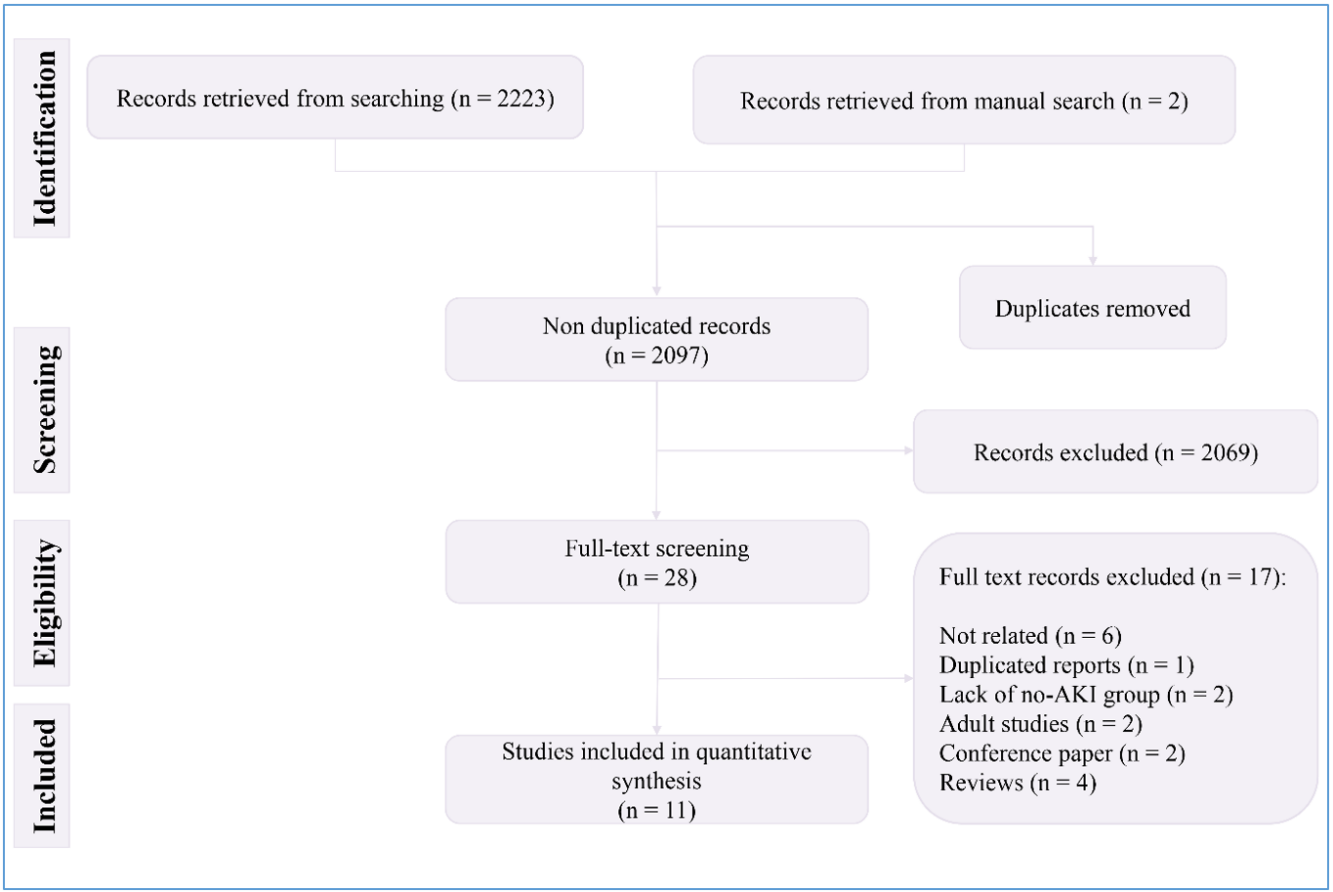
PRISMA flow diagram of present meta-analysis

The literature search yielded 2223 records, 2097 of which were non-duplicates. After the initial screening, full texts of 28 articles were studied in detail and finally, the data of 11 articles were included in the present study (15–25) (Figure 1). All studies were cohorts. One article contained data from four separate cohorts (15). Therefore, the data of each cohort were reported separately. Three retrospective cohorts and 11 prospective cohorts were included in the present study. The studied patients were ICU admitted in 12 cohorts. These studies included data from 3701 children (60.41% boys). There were 752 children with AKI and 2949 non-AKI children. The severity of AKI was severe in 12 cohorts. All studies had assessed RAI status at the time of admission and used a cut-off point of 8 to predict AKI. Patients were followed up for 3 days in 13 cohorts and until discharge in one cohort. Table 1 shows the characteristics of the included studies.

- **Risk of bias and publication bias across studies**

**Figure 2:**
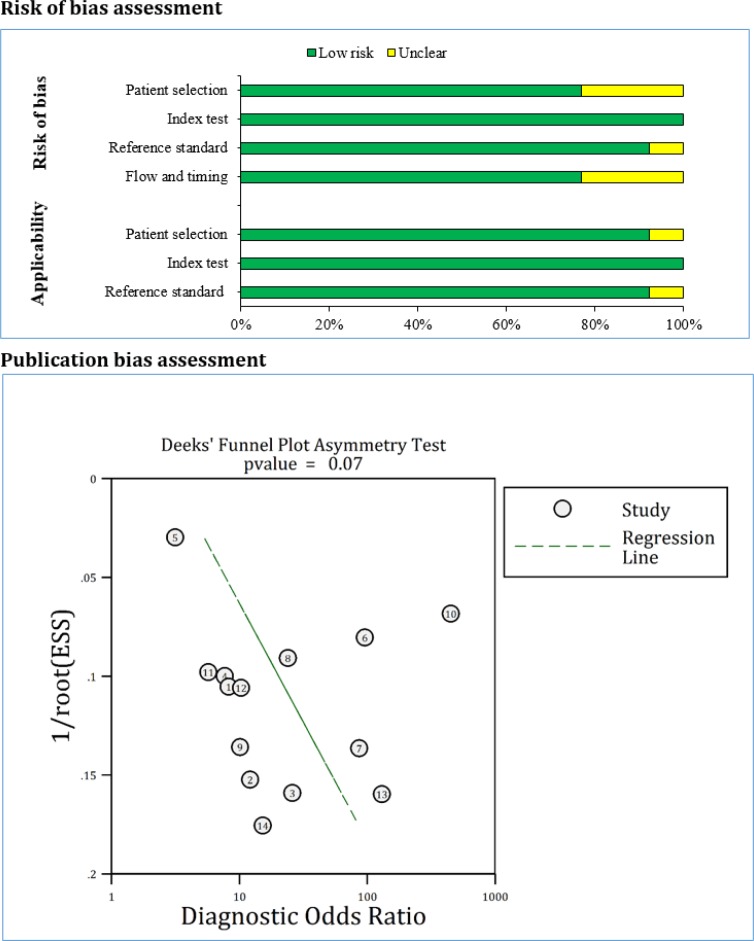
Risk of bias and publication bias assessments. There is no evidence

Risk of bias assessment based on the QUADAS-2 guidelines showed that the risk of bias in patient selections and flow and timing was unclear in three cohorts. The risk of bias in reference index was unclear in one cohort. Also, applicability of patient selection and reference standard was unclear in one cohort. Finally, analysis showed that there is no evidence for publication bias in the present study (p = 0.074) (Figure 2).

- **Discriminatory power of RAI in AKI**

**Figure 3:**
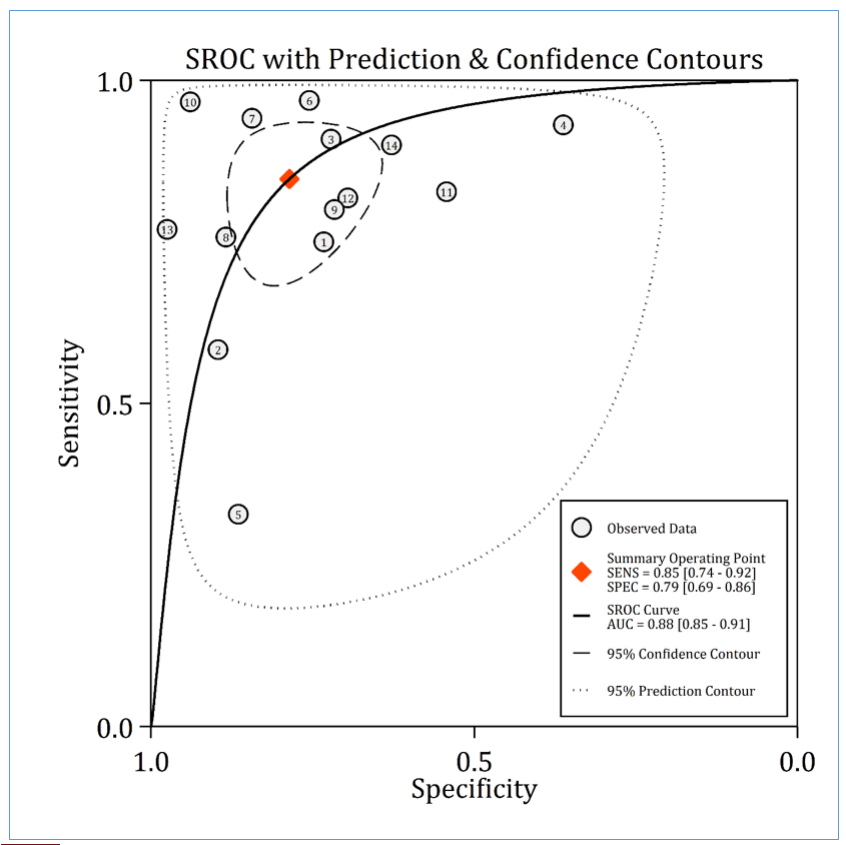
Area under the summary receiver operative characteristics (SROC) curve (AUC). SENS: Sensitivity; SPEC: Specificity

**Figure 4:**
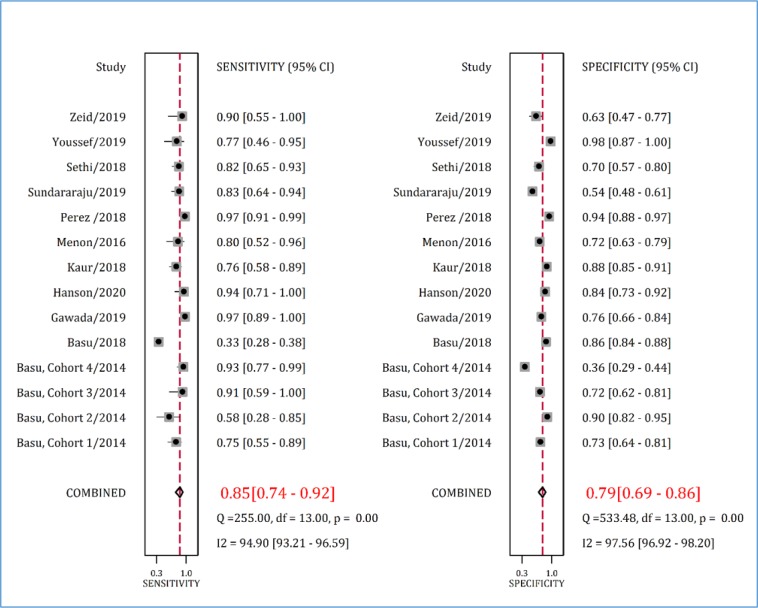
Sensitivity and specificity of renal angina index in prediction of acute kidney injury. CI: Confidence interval

Pooled analysis showed that the AUC of RAI in paediatric AKI prediction was 0.88 (95% CI: 0.85 to 0.91) (Figure 3). Sensitivity and specificity of this tool in predicting AKI were 0.85% (95% CI: 0.74% to 0.92%) and 0.79% (95% CI: 0.69% to 0.89%), respectively (Figure 4).

**Figure 5:**
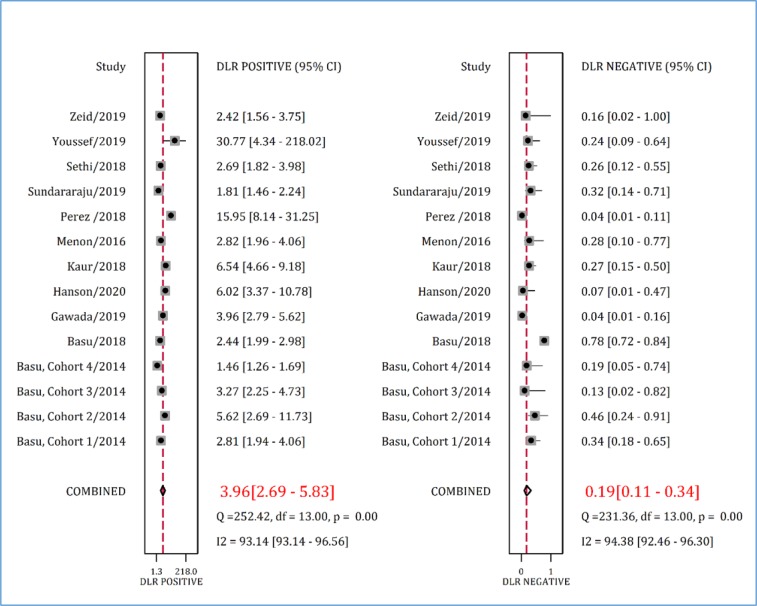
Positive and negative diagnostic likelihood ratios (DLR) of renal angina index in prediction of acute kidney injury. CI: Confidence interval

**Figure 6:**
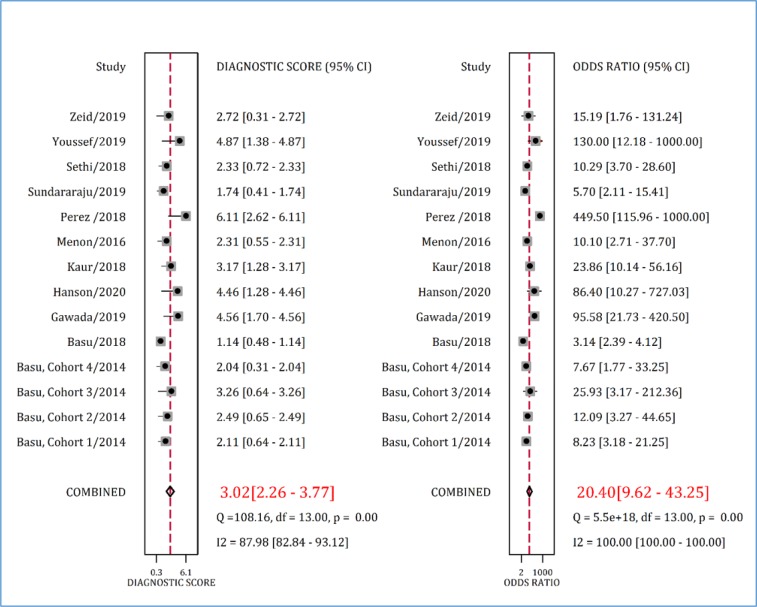
Diagnostic score and diagnostic odds ratio of renal angina index in prediction of acute kidney injury. CI: Confidence interval

Positive and negative likelihood ratio of RAI in predicting AKI in children were 3.96 (95% CI: 2.69 to 5.83) and 0.19 (95% CI: 0.11 to 0.34), respectively (Figure 5). Finally, the diagnostic odds ratio of RAI was 20.40 (95% CI: 9.62 to 43.25) (Figure 6).

## Discussion

Early detection of AKI in children can prevent persistent kidney damage such as chronic kidney failure and end-stage renal disease. For this purpose, the present meta-analysis examined the discriminatory power of RAI in predicting AKI in children on admission. The findings showed that RAI is a reliable tool in predicting AKI in children.

Diagnostic odds ratio of RAI in predicting AKI was about 20, indicating its high applicability in management of the patients. However, there were 7.94% false negatives for RAI. Although RAI has high discriminatory precision, we should consider a proper solutions to reduce its false negative rate. One of these solutions is to add other biomarkers to the RAI model. In this regard, a study has shown that adding Syndecan-1 to RAI increases its predictive value (26). In another study, Basu et al. showed that the addition of any of plasma neutrophil gelatinase-associated lipocalin (NGAL), matrix metalloproteinase-8 (MMP-8), and neutrophil elastase-2 (Ela-2) biomarkers increased the discriminatory power of RAI (27). However, further studies are still needed to investigate the cost-effectiveness of adding a new biomarker to RAI.

The lowest sensitivity for RAI was reported in the study by the Basu et al. This multicenter study had the largest sample size among the included studies. The sensitivity and specificity of RAI in this study were 33% and 86%, respectively (16). The findings of this study may be outliers. We performed an additional analysis after excluding the Basu et al. article. The findings showed that omitting this article did not have a significant effect on the reported sensitivity (0.87 vs. 0.85) and specificity (0.78 vs. 0.79) of RAI in detection of AKI. 

In the present meta-analysis, 14 cohort studies (from 11 articles) were included, 3 of which were retrospective and 11 were prospective. The retrospective nature of these studies partly influenced the quality of the published articles and led to an unclear risk of bias in patient selection. In addition, one study (21) did not indicate the reference index used for classification of children to AKI and non-AKI. Therefore, the status of this study in the reference index section was unclear. However, the risk of bias and applicability of most studies were low, which is a strong point for the present study.

**Table 1:** Characteristics of included studies

**Author; year; ** **Country**	**Study type**	**Setting of patients**	**Age**	**No. AKI; non-AKI**	**No. boys**	**AKI definition**	**AKI severity**	**RAI time; cut-offs**	**Follow up **	**TP**	**FP**	**TN**	**FN**
Basu, ***Cohort 1***; 2014; USA	RCS	Sepsis	0.2 to 12.5	28; 116	83	KDIGO	Severe	0; 8	3	21	31	85	7
Basu, ***Cohort 2***; 2014; Canada	RCS	ICU admitted	0.2 to 12.5	12; 106	74	KDIGO	Severe	0; 8	3	7	11	95	5
Basu, ***Cohort 3***; 2014; Canada	PCS	ICU admitted	0.2 to 12.5	11; 97	64	KDIGO	Severe	0; 8	3	10	27	70	1
Basu, ***Cohort 4***; 2014; USA	PCS	ICU admitted	0.2 to 12.5	29; 185	134	KDIGO	Severe	0; 8	3	27	118	67	2
Basu; 2018; Multicenter	PCS	ICU admitted	2 to 14.5	553; 1037	882	KDIGO	Severe	0; 8	3	121	165	1057	247
Gawada; 2019; India	PCS	ICU admitted	0.1 to 12	114; 48	95	KDIGO	Severe	0; 8	3	62	24	74	2
Hanson; 2020; USA	PCS	ICU admitted	0.1 to 25	17; 64	38	KDIGO	Any AKI	0; 8	in-hospital	16	10	54	1
Kaur; 2018; India	PCS	ICU admitted	0.1 to 18	53; 360	301	KDIGO	Severe	0; 8	3	25	44	336	8
Menon; 2016; USA	PCS	ICU admitted	0.2 to 25	15; 141	98	KDIGO	Severe	0; 8	3	12	40	101	3
Perez; 2018; Philippine	RCS	Sepsis	<19	90; 132	130	NR	NR	0; 8	3	87	8	124	3
Sethi; 2018; India	PCS	ICU admitted	6.5 ± 5.9 months	33; 69	69	KDIGO	Severe	0; 8	3	27	21	48	6
Sundararaju; 2019; India	PCS	ICU admitted	0.1 to 18	29/256	189	KDIGO	Severe	0; 8	3	24	117	139	5
Youssef; 2019; Egypt	PCS	ICU admitted	0.2 to 14	13; 40	34	pRIFLE	Severe	0; 8	3	10	1	39	3
Zeid; 2019; Egypt	PCS	ICU admitted	0.2 to 7	10; 43	45	pRIFLE	Severe	0; 8	3	9	16	27	1

AKI: Acute kidney injury; FN: False negative; FP: False positive; KDIGO: Kidney Disease Improving Global Outcomes; NR: Not reported; PCS: Prospective cohort study; pRIFLE: Pediatric Risk, Injury, Failure, Loss, End Stage Renal Disease; RCS: Retrospective cohort study; TN: True negative; TP: True positive; ICU: Intensive care unit.

**Table 2:** Risk of bias assessment

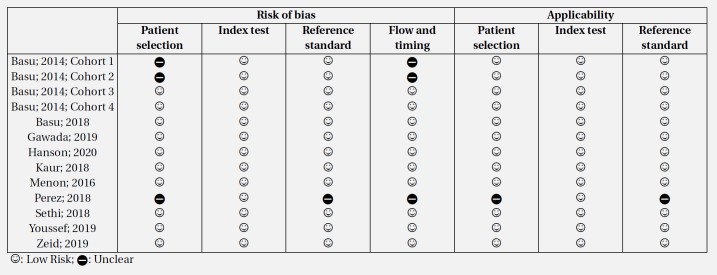

## Conclusion:

The present meta-analysis summarized evidence on the discriminatory power of RAI at the time of admission in predicting AKI in children and adolescents. The findings of this study showed that RAI is a reliable tool in predicting AKI in children.
